# The complete chloroplast genome of an economic plant, *Camellia sinensis* cultivar Anhua, China

**DOI:** 10.1080/23802359.2018.1462124

**Published:** 2018-05-10

**Authors:** Meng Dong, Shiquan Liu, Zhenggang Xu, Zhiyuan Hu, Wenzhen Ku, Liang Wu

**Affiliations:** a Hunan Provincial Key Lab of Dark Tea and Jin-hua, School of Materials and Chemical Engineering, Hunan City university, Yiyang, China;; b Hunan Research Center of Engineering Technology for Utilization of Environmental and Resources Plant, Central South University of Forestry and Technology, Changsha, China

**Keywords:** Chloroplast genome, *Camellia sinensis*, dark tea, Illumina, Sequencing

## Abstract

In order to supply genetic information of *Camellia sinensis* cultivar Anhua, characterization of the complete chloroplast genome sequence was reported based on high-throughput sequencing data. The complete cp genome of *C. sinensis* cultivar Anhua is shorter than other *C. sinensis* cultivars with 157,025 bp in length, comprising a large single copy (LSC) region of 86,585 bp and a small single copy (SSC) region of 18,276 bp, separated by two inverted repeat regions (IRs) of 26,082 bp. The overall G + C content is 37.30%. The genome contained total of 135 genes, including 90 protein coding genes, 37 tRNA genes, and 8 rRNA genes. Phylogenetic analysis showed all the cultivars were clustered into a group except *C. sinensis* var. pubilimba.

*Camellia sinensis* cultivar Anhua, which belongs to Theaceae, is evergreen small tree or shrub. Leaf of the economic plant is the major source of dark tea, which becomes increasingly popular owing to its special health benefits. In accordance with dark tea production area, *C. sinensis* is cultivated mainly in Hunan, Sichuan, and Yunnan provinces in China (Zheng et al. 2015). The quality of dark tea have close relationship with cultivar of *C. sinensis* and geographical teas are formed (Ning et al. 2016). Because of the special planting environment, *C. sinensis* cultivar Anhua is famous and Anhua brick dark tea sell well (Chen et al. 2015). Except for materials of dark tea, other economic functions are explored based on the rich secondary metabolites (Shen et al. 2018). Tea cultivation has a long history and there are so many cultivar populations. Genetic diversity of *C. sinensis* cultivars were revealed in many researches (Roy and Chakraborty 2009; Yuan et al. 2015). Lots of germplasm resources of *C. sinensis* were lost gradually due to the long period artificial selection. Thus, it is urgent and necessary to protect and make use of *C. sinensis,* the germplasm resources. In this study, characterization of the complete chloroplast genome sequence of *C. sinensis* cultivar Anhua was reported based on high-throughput sequencing data with the accession number MH042531.

Fresh leaves of *C. sinensis* cultivar Anhua were collected from an individual in plant garden of Hunan City university (Hunan, China; N28°32′35.76″, E112°22′53.53″) and the samples were kept in the laboratory at −80 °C under the accession number 20171121DM. The whole genomic DNA was extracted from fresh leaves, using DNeasy Plant Mini Kit (QIAGEN,Hilden, Germany).Total chloroplast DNA was extracted with Plant Chloroplast Purification Kit (BTN120308, Beijing, China) and Column Plant DNA Extraction Kit(K183001, Massachusetts, USA) and sequenced by Illumina Miseq Platform (California, USA). Filtration and annotation of the whole genome and drawing of a circular map referred to Zhang’s method by using Trimmomatic v0.32, DOGMA and cpGAVAS, OGDRAW (Wyman et al. 2004; Lohse et al. 2007; Yong et al. 2012; Bolger et al. 2014; Zhang et al. 2017).

The complete cp genome of *C. sinensis* cultivar Anhua is shorter than other *C. sinensis* cultivars (KJ806281, KJ806279, KC143082, NC020019, KJ996106, KJ806280, KF562708) with 157,025 bp in length, comprising of a large single copy (LSC) region of 86,585 bp and a small single copy (SSC) region of 18,276 bp, separated by two inverted repeat regions (IRs) of 26,082 bp. The overall G + C content is 37.30%. The genome contained total of 135 genes, including 90 protein coding genes, 37 tRNA genes, and 8 rRNA genes. Among the protein coding genes, *atpF*, *rpoC1*, *rpl2*, *ndhB*, *ndhA*, *ycf1* contained one intron, *ycf3* and *clpP* included a couple of introns.

A molecular phylogenetic tree was constructed with the MEGA7.0 to confirm phylogenetic position of *C. sinensis* cultivar Anhua (Kumar et al. 2016) based on all common protein coding genes of other cultivars and species from different genus (Figure 1). The result showed all the cultivars were clustered into a group except *C. sinensis* var. pubilimba. A good foundation for population genomic studies and genetic engineering was laid through this annotated chloroplast genome.

**Figure 1.**
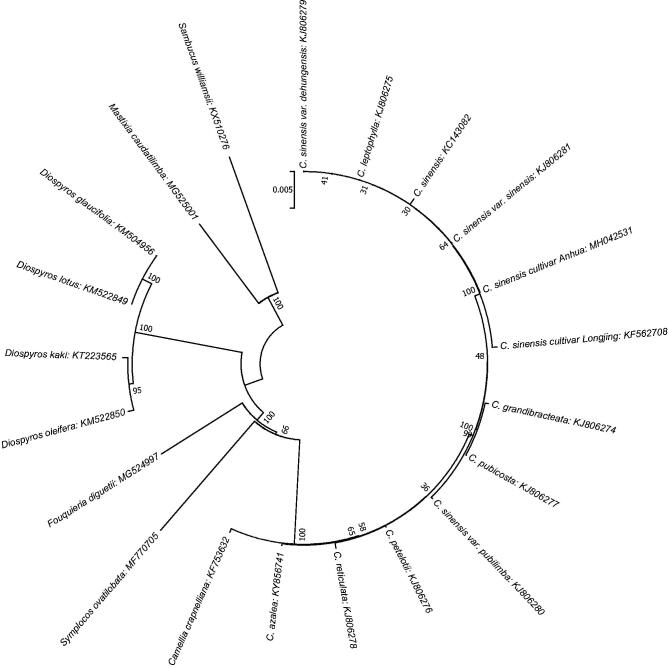
The phylogenetic tree based on 21 complete chloroplast genome sequences. The neighbour-joining (NJ) phylogenetic tree was constructed with MEGA 7 (with 1000 bootstrap replicates).
